# Malignant Peripheral Nerve Sheath Tumor of the Small Bowel: An Unusual Presentation with Fatal Outcome

**DOI:** 10.1155/2013/423867

**Published:** 2013-08-28

**Authors:** Amina Mohtaram, Siham Mesmoudi, Hind M'rabti, Amal Rami, Rachida Latib, Zakia Bernoussi, Imane Aaribi, Meryam Ben Ameur El Youbi, Hassan Errihani

**Affiliations:** ^1^Department of Medical Oncology, National Institute of Oncology, Rabat, Morocco; ^2^Department of Pathology, Centre Hospitalier IBN SINA, Rabat, Morocco; ^3^Department of Radiology, National Institute of Oncology, Rabat, Morocco

## Abstract

Malignant peripheral nerve sheath tumor of the small bowel is an extremely rare disease. Histologic distinction from other types of soft tissue sarcoma especially fibrosarcoma and leiomyosarcoma requires electron microscopy. Complete surgery remains the only curative treatment. However, late diagnosis makes curative surgery more difficult. The contribution of chemotherapy to incomplete surgery has been proved without controlled studies. We report a case of this type of lesion discovered following a small bowel perforation.

## 1. Introduction

Malignant peripheral nerve sheath tumors are defined as any tumor arising from a peripheral nerve or exhibiting nerve sheath differentiation. The incidence of malignant peripheral nerve sheath tumors is 1/100 000, corresponding to 3–12% of soft tissue sarcomas [1]. They are commonly arising on the trunk, extremities, head and neck, and paravertebral region [2].

Malignant peripheral nerve sheath tumors arising from the gastrointestinal tract are extremely rare, with a few cases being reported in the literature for the small bowel [2, 3]. Because the clinical symptoms in this localization are usually nonspecific, the diagnostic is often late and makes wide excision more difficult. In this report, we describe a malignant peripheral nerve sheath tumors of small bowel discovered following an intestinal perforation.

## 2. Case Report

A 18-year-old patient without pathological antecedents especially neurofibromatosis presented with abdominal pain associated with diarrhea and vomiting. The clinical examination revealed an abdominal defence. The patient underwent emergency surgery for peritonitis.

 At laparotomy, the surgeon discovered a tumour in the proximal jejunum associated with peritonitis in the right hypochondrium and carcinomatosis implants of the colon, omentum, and mesentery. Tumor excision with 40 cm resection of the small intestine was performed. Macroscopic examination showed a 40 cm resected small bowel, containing a tumor measuring 20 × 19 × 12 with parietal development. The tumor had a gray-white appearance with small cysts and a few areas of hemorrhage. 

**Figure 1:**
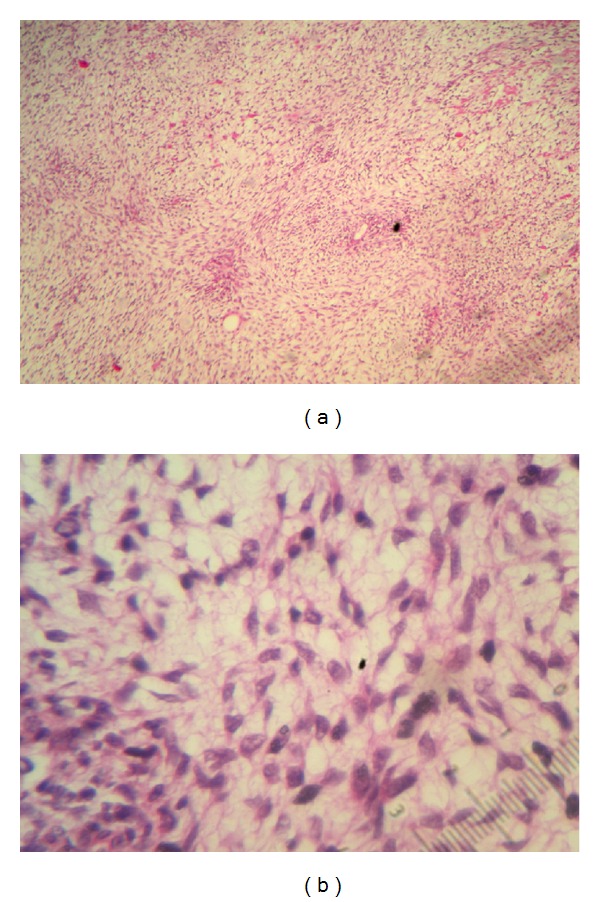
Microscopic findings of small bowel showing (a) cellular spindle cell tumor in fascicles with (b) cytonuclear atypia and high mitotic index (HE ×40).

Histologically, the tumor showed malignant spindle cells. The cytoplasm was scant abundant with anisokaryosis. The mitotic activity was 9 mitoses per 10 high-power fields. Myxoid stroma reaction was found, and there were negative surgical margins (Figure 1).

**Figure 2:**
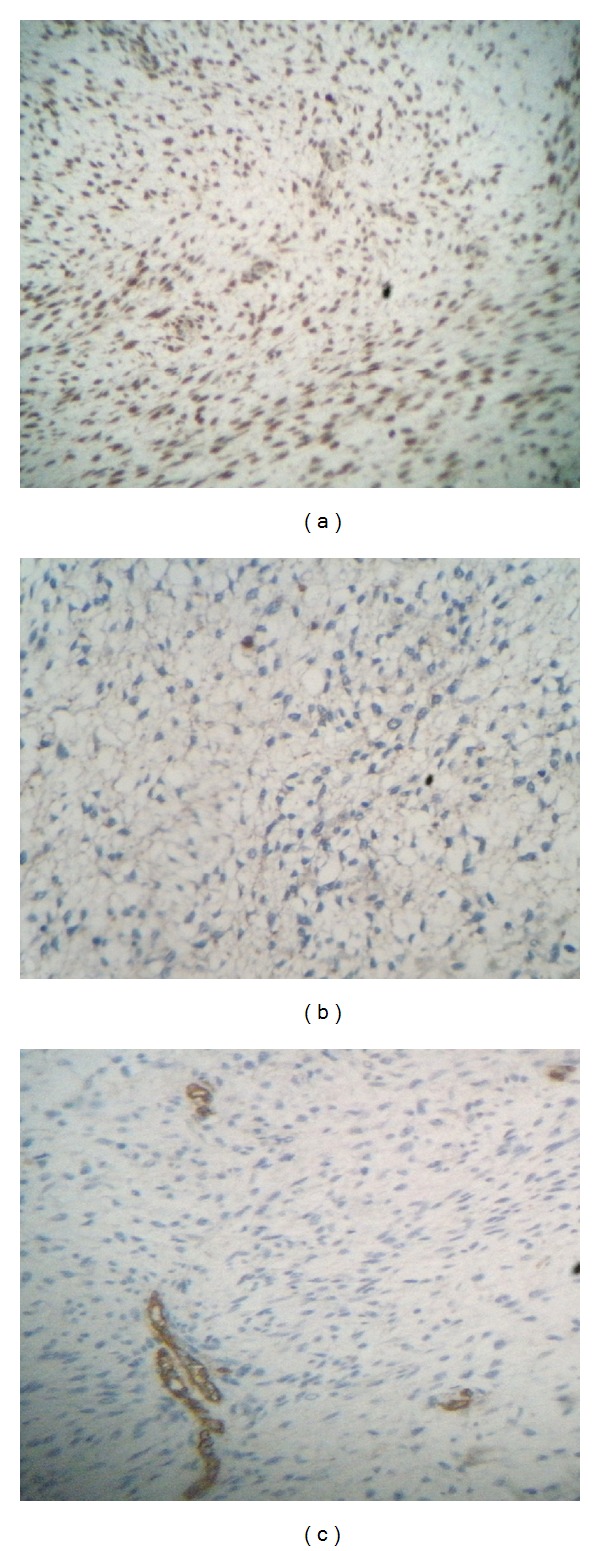
Immunohistochemical analysis demonstrating positive staining for S-100 protein (a) and negative staining for CD117 (b) and CD34 (c).

On immunohistochemical staining, the tumor was negative for CD34, CD117, and smooth muscle actin but positive for S100 protein. The Ki67 proliferation index was higher at 60%. Based on these findings, the tumor was identified as a malignant peripheral nerve sheath tumor (Figure 2).

**Figure 3:**
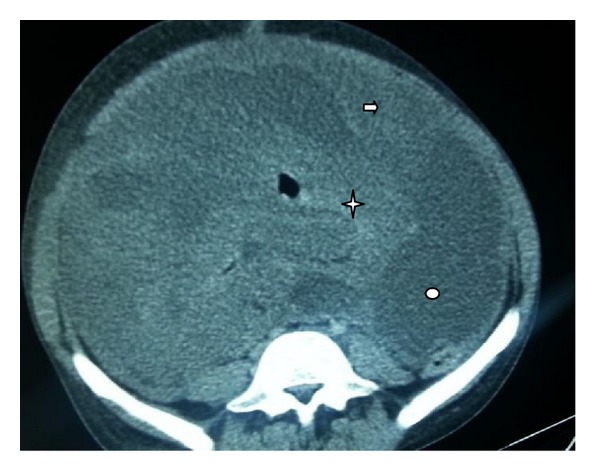
Computed tomography scan of abdomen showing an important thickening of small bowel (star), with ascites (oval) and nodules of peritoneal carcinomatosis (arrow).

The postoperative computed tomography showed a diffuse thickening of small bowel with ascites and nodules of peritoneal carcinomatosis without distant metastases (Figure 3). 

Biochemical analysis was normal. The patient died by his disease before undergoing chemotherapy.

## 3. Discussion

Malignant peripheral nerve sheath tumor is a rare variety of soft tissue sarcoma of ectomesenchymal origin. The World Health Organization introduced the term malignant peripheral nerve sheath tumor, and this replaced previous heterogeneous and often confusing terminology of malignant schwannoma, neurilemomma, and neurofibrosarcoma [1–4].

Malignant peripheral nerve sheath tumor may arise spontaneously in adult patients in the third to sixth decade of life, although from 5% to 42% of these tumors have an association with type-I multiple neurofibromatosis [5]. Our patient was young and did not suffer from this autosomal dominant disorder disease. 

The clinical symptoms of these tumors of the gastrointestinal tract are nonspecific, including abdominal pain (63%), emesis (43%), weight loss (44%), and gastrointestinal bleeding (23%) [6]. 

Histologic diagnosis of  malignant peripheral nerve sheath tumor is actually facilitated by the presence of a palisading arrangement, bizarre giant cells, nuclear atypia, mitotic figures, and necrosis. These tumors have morphological heterogeneity, and staining analysis reveals highly cellular spindle cell tumor in fascicles [7]. There are no specific histological or immunohistochemical markers for malignant peripheral nerve sheath tumor. S-100 protein is highly characteristic of neural-derived neoplasms. However, S100 protein immunoreactivity is detected in only 50–60% of malignant peripheral nerve sheath tumor and is also expressed in a range of other tissues and tumor types [8, 9]. Different markers are used to exclude other spindle cell tumors. Desmin and *α*-SMA are used to exclude smooth muscle tumors, and CD34 and CD117 (c-kit) are used to exclude GIST [10]. High levels of P53 and Ki67 may also be related to malignant peripheral nerve sheath tumor [11]. In our case, the strong S-100 expression without expression of other immunohistochemical markers and high levels of Ki67 was in favor of malignant peripheral nerve sheath tumor.

The prognosis for malignant peripheral nerve sheath tumor of the small bowel remains unknown. A recently published study investigated the overall prognostic factors and survival of patients with malignant peripheral nerve sheath tumor in all locations [12, 13]. The study suggested that the disease-specific survival rate at 10 years was 31.6% for 87 primary disease patients, 25.9% for 26 recurrent patients, and 7.5% for 27 metastatic patients after median followup of 91 months. The strongest independent predictors of survival were primary versus recurrent disease, tumor size, tumor site, and margin status [12]. 

To date, treatment is unknown for malignant peripheral nerve sheath tumor arising from the small bowel. So, current recommendations may be based only on what is known for this tumor in other locations of the body [3]. 

Radical surgical treatment with wide excision for malignant peripheral nerve sheath tumor is the treatment of choice. Most case series indicate limited benefits and high morbidity of using adjuvant radiotherapy or chemotherapy. These treatments should be reserved for cases of positive margins, recurrent disease, or when wide local excision is unfeasible [2, 3].

Because of the rarity of malignant peripheral nerve sheath tumor, there is no phase II or III trials assessing the role of chemotherapy in unresectable and metastatic tumors. In a retrospective series, doxorubicin-ifosfamide combination was associated with a better outcome with a median progression-free survival of 26.9 weeks (range 22.4–35.1), justifying further investigations of this combination [14]. In our case, the patient died before undergoing chemotherapy.

## 4. Conclusion

We report here a rare localization of malignant peripheral nerve sheath tumor in the small bowel. Diagnosis and treatment of those tumors require a multidisciplinary approach. Based on our case and those of the literature, the diagnosis is often delayed because of nonspecific symptoms. There is no standard treatment for malignant peripheral nerve sheath tumor in the small bowel. After reviewing the literature, the treatment is based on that of soft tissue sarcomas in other locations. Chemotherapy in advanced tumors is the treatment of choice.
